# Fungal infection of the central nervous system: Autopsy analysis of six cases

**DOI:** 10.1177/2050313X221122419

**Published:** 2022-09-09

**Authors:** Moshawa Calvin Khaba, Tshepo Cletus Ngale, Ndivhuho Agnes Makhado

**Affiliations:** Sefako Makgatho Health Sciences University, Pretoria, South Africa

**Keywords:** Fungal infection, central nervous system, autopsy, pathology, immunosuppression

## Abstract

Fungal infections of the central nervous system are fatal and rare clinical entities observable in immunosuppressed patients from varying causes. They carry higher risks of morbidities and mortality as compared to viral, bacterial or parasitic central nervous system infections. This study describes clinicopathological description of the central nervous system fungal infections with antemortem diagnostic challenges. This is a 9-year retrospective study of six cases composed of three females and three males with a mean age of 29.3 years. All six decedents presented with signs of meningeal irritation. They all suffered from immunodeficiency of varying causes. The gross and microscopic features revealed cryptococcosis, candidiasis and mucormycosis as the cause of the central nervous system infection. Early diagnosis and appropriate medical treatment are of paramount importance in improving the overall survival of patients with central nervous system mycosis. A few autopsy cases with fungal infection of the central nervous system have been described; therefore, more autopsies studies are needed to re-enforce on the existing epidemiology of these fatal infections. Moreover, this will assist in further elucidating the varying gross features and tissue reaction patterns associated with them.

## Introduction

Fungal infection of the central nervous system (CNS) is usually a disease of immune-compromised patients from HIV infection, post-transplantation immune-suppression, corticosteroid use and cancer chemotherapy.^1^ The most common isolated organisms are *Cryptococcus neoformans, Candida* spp. and *Aspergillus* spp. while mucorales are less frequent.^1^ The intracranial seedlings occur either during haematogenous spread from other sites or direct extension from the juxta-cranial sites which can happen during neurosurgical procedures.^2^ The clinical presentation ranges from meningoencephalitis, brain abscess and infarction to stroke.^3,4^ These infections are highly fatal despite available treatment, and this may be due to their diagnostic conundrum.^1,3,5^ Therefore, thorough history taking and clinical examination with appropriate investigations increase the high index of suspicion of these infections.^6^ Herein, we present six autopsy cases in which there were clinical challenges in the diagnosis, and therefore, providing suboptimal management which led to the deaths of these individuals.

## Material and methods

A case series consisting of six autopsy cases with the final diagnosis of fungal infection of the CNS was conducted from 1 January 2013 to 31 August 2021. The clinicopathologic characteristics such as age, sex, co-morbidities including HIV status, clinical presentation and antemortem diagnosis, mycology and treatment information were retrieved from the laboratory information system (LIS) as well as hospital medical records. Gross descriptions of the organs were reviewed based on archived photography where available. Haematoxylin and eosin (H&E)–stained sections, Gomori’s methenamine silver (Grocott’s), Fontana Masson, Periodic Acid–Schiff (PAS) and Mucicarmine histochemical stains were re-appraised to confirm the fungi using standard histomorphological diagnostic criteria.

## Results

### Clinical features

**Figure 1.**
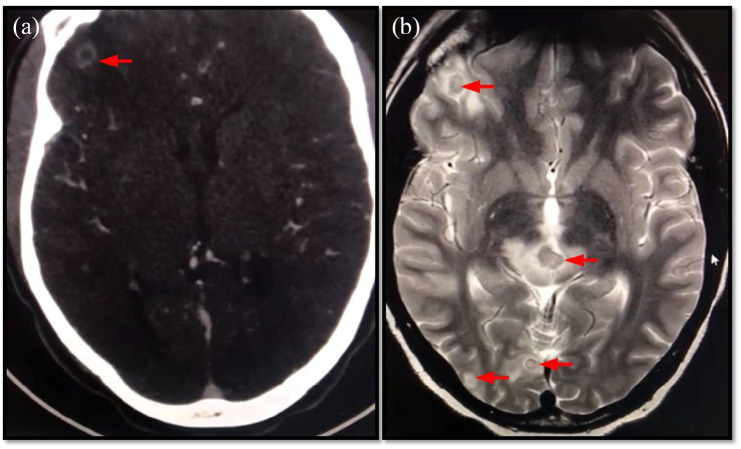
(a) CT brain showing ring-enhancing lesion on the left frontal lobe. (b) MRI showing multiple circumscribed in the pons, occipital and frontotemporal lobes (red arrows).

The study consisted of six decedents equally distributed among males and females, with a mean age of 29.3 years (5–49) (Table 1). The common presenting symptom was headache (5/6). It was accompanied by the following symptoms: vomiting (2/5), dizziness (1/5), blurred vision (1/5), fever (1/5), fatigue (1/5) and cough (1/5). In the single case where headache was not reported, the decedent presented with fatigue, weight loss and diarrhoea. A combination of the symptoms of meningeal irritation (headache, vomiting, dizziness and neck stiffness) was only present in case 2. The presence of headache, vomiting and fever in case 6 could be highly attributable to meningitis. A computed tomography (CT) scan and a magnetic resonance imaging (MRI) of the brain were only performed in case 4 and showed multiple lesions in the pons, occipital and frontotemporal lobes (Figure 1(a)–(b)).

**Table 1.** Clinicopathological features.

Clinicopathological features	Case 1	Case 2	Case 3	Case 4	Case 5	Case 6
Age (years)	49	40	36	29	17	5
Sex	Male	Male	Female	Female	Male	Female
Co-morbidities/predisposing factors	Unknown. Stigmata of HIV infection	HIV positive	HIV positive	Myasthenia gravis on steroid therapy	Neurosurgical intervention	Nephroblastoma stage V, chemotherapy
Clinical presentation	Fatigue, weight loss and diarrhoea	Headache, vomiting, dizziness and neck stiffness	Headache, blurred vision, productive cough with constitutional symptoms	Headache, mild cough, fatigue	Headache, vomiting	Renal failure, headache, vomiting and fever
Antemortem diagnosis	Tuberculosis	Crytococcal meningitis	Tuberculosis cryptococcal meningitis	CNS tuberculoma or metastatic disease	Hydrocephalus	Septicaemia
Gross						
Brain	Increased weight and size. Dusky surface. Features of raised intracranial pressure with tonsillar herniation	Increased weight and size. Dusky surface. Multiple cystic spaces in the basal ganglia and dentate nucleus. Necrosis of the anterior commissure	Increased weight and size. Dusky surface. Multiple cysts in the thalamus. Necrosis of anterior commissure	Normal weight. Multiple circumscribe lesions on the pons, hippocampus, frontotemporal areas	Increased weight and size with features of raised intracranial pressure. Exudate within the sagittal sinus and lateral sulci	Increased weight and size with raised intracranial pressure
Microscopy						
Diagnosis	Cryptococcosis	Cryptococcosis	Cryptococcosis	Cryptococcosis	Candidiasis	Mucormycosis
H&E	Soap bubble appearance. No inflammatory reaction	Soap bubble appearance. No inflammatory reaction	Soap bubble appearance. No inflammatory reaction	Soap bubble appearance. No inflammatory reaction	Granulomatous inflammation, leukocytoclastic vasculitis and thrombosed vessels	Necrosis and angioinvasion
Other organs involved	Lungs, kidneys, liver, spleen, thyroid, lymph nodes	Lungs, kidneys, liver, oesophagus	Lungs, liver, kidney	Lung, heart	Lung, heart	Lung, heart, liver, kidney

CNS: central nervous system; H&E: haematoxylin and eosin.

Five decedents had immunosuppression from varying causes which ranged from human immunodeficiency virus (HIV)/acquired immunodeficiency syndrome (AIDS) (2/6), post-chemotherapy from malignant tumour (1/6) to myasthenia Gravis (MG) on steroid therapy (1/6). One decedent refused to be tested for HIV, but he had clinical stigmata of retroviral disease. Another decedent had neurosurgical intervention and prolonged antibiotic as predisposing factors.

The differential diagnosis of the six cases was as follows: tuberculosis (3/6), cryptococcal infection (2/6), septicaemia (1/6), hydrocephalus (1/6) and metastatic malignancy (1/6).

The decedent in case 1 was correctly diagnosed with cryptococcal meningitis and started on antifungal therapy. The decedent in case 2 was suspected to have cryptococcal meningitis; however, treatment could not be commenced as the decedent died in the emergency room. The correct antemortem diagnosis could not be established on decedents in cases 3–6.

### Pathological findings

Complete autopsies were performed on all six cases with meticulous gross examination of each organ. However, for the purpose of this study, special emphasis was on the CNS organs (brain and meninges).

**Figure 2.**
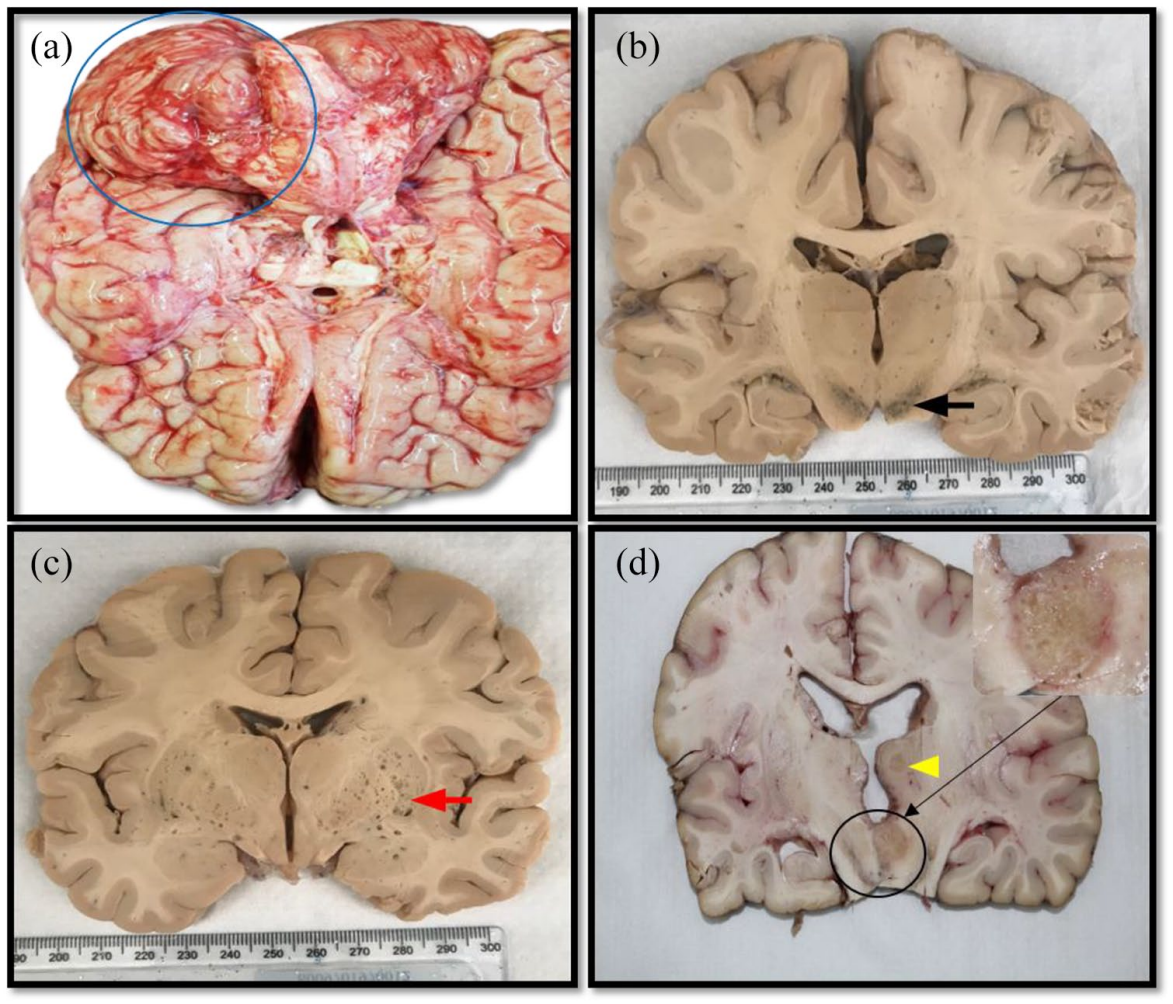
Brain (gross) in cryptococcosis. (a) Features of raised intracranial pressure and tonsillar herniation, (b) necrosis of the thalamus (black arrow), (c) microcysts involving the thalamus (red arrow) and (d) multiple circumscribed lesions on the pons (yellow arrow head).

Grossly, the average brain weight was 1337 g in all six cases. Case 1, 5 and 6 showed features of raised intracranial pressure. Only case 1 had associated tonsillar herniation (Figure 2(a)). Furthermore, in case 5, there was exudate on the brain surface. The brain cut surface in cases 2 and 3 revealed cystic spaces with gelatinous contents (Figure 2(b) and (c)) while case 4 showed multiple friable lesions on the midbrain and frontotemporal lobe (Figure 2(d)).

**Figure 3.**
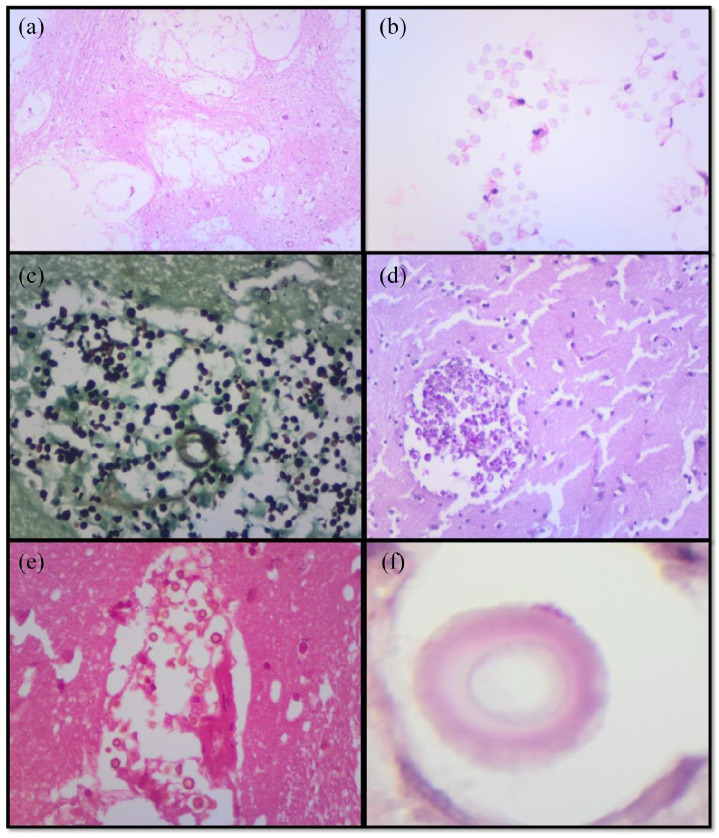
(a–f) Cryptococcal infection. (a) Soap bubble appearance and (b) cryptococcal organism. (c and d) Histochemical stains highlight fungal organisms: (c) Grocott and (d) PAS. (e) Fontana Masson highlights melanin pigment in the cell wall and (f) mucicarmine highlights the capsule.

**Figure 4.**
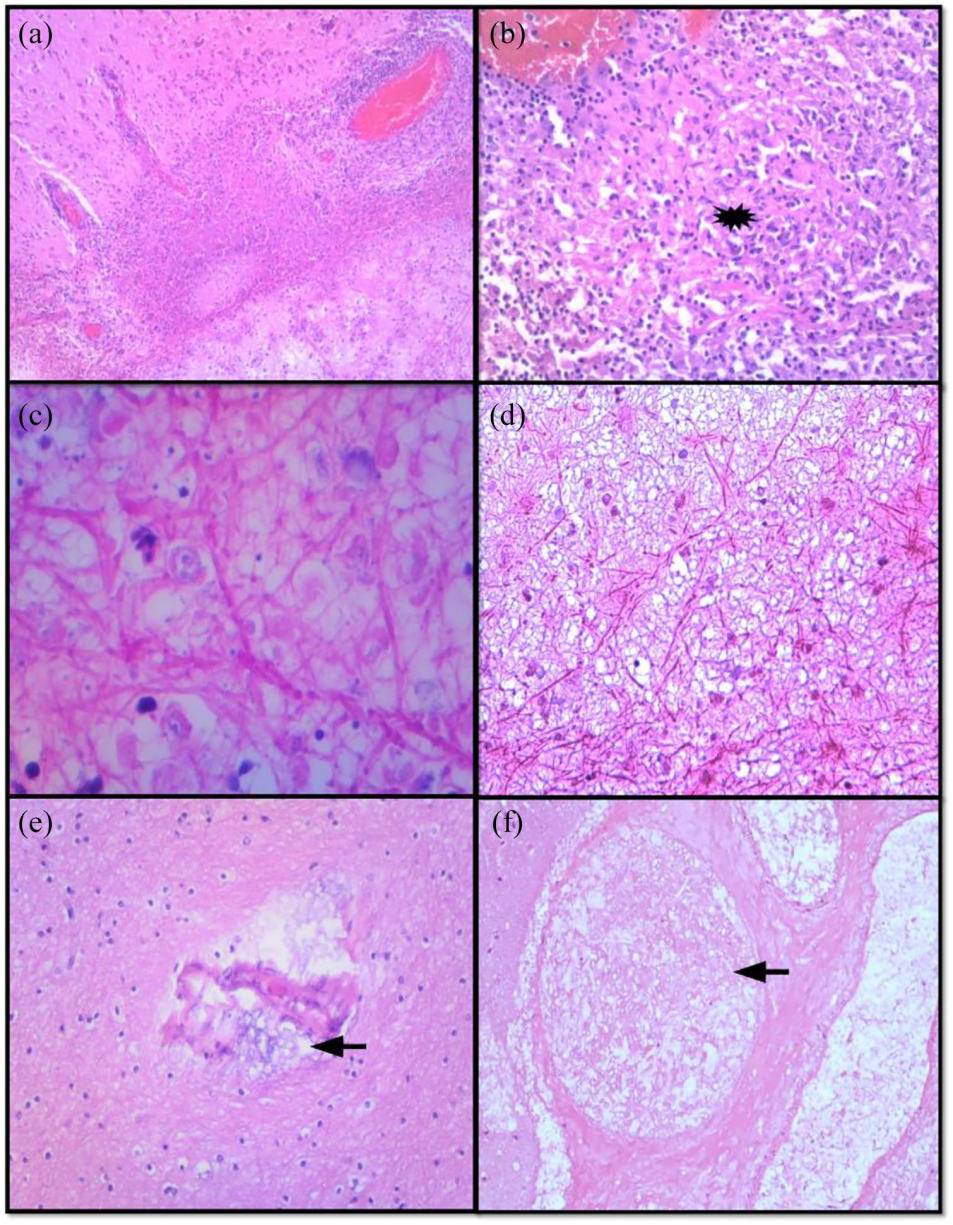
(a)–(d) Candidiasis. (a) Acute inflammation and (b) granulomatous inflammation. (c and d) Candida pseudohyphae and yeasts. (e and f) Mucormycosis with angioinvasion (black arrow).

Microscopically, four cases (1–4) showed cryptococcal yeast without inflammatory response. The fungal yeasts were pleomorphic with narrow neck budding which imparted a ‘soap bubble appearance’ (Figure 3(a)). Grocott and PAS highlighted the fungi, Fontana Masson showed the melanin pigment in the cell wall while Southgate’s mucicarmine confirmed the capsule. Case 5 showed candida hyphae with non-necrotising granulomatous response, vasculitis and thrombosed vessels (Figure 4(a)–(d)). Mycobacterial infection was excluded by the negative Ziehl–Neelsen stain. The PAS stain highlighted the candida hyphae. Case 6 confirmed mucormycosis with associated necrosis and angioinvasion. The fungal hyphae, which were also highlighted by PAS and Grocott, were wide, aseptate with right angle branching (Figure 4(e) and (f)).

## Discussion

In general, fungal infections are not notifiable diseases, and the precise information on their prevalence throughout the world is not available.^4^

The incidence of fungal infection of the CNS as the cause of death from autopsy in South Africa is likely an under-representation of these infections as the number of autopsies performed in the modern era has decreased. Furthermore, the bulk of cases is assigned cause of death based on the clinical impression from hospitals and/or home, which contributes to further under-reporting. The high prevalence of HIV infection in the area has also made it easier for clinicians to attribute the cause of death in this population to tuberculosis, which can be indistinguishable to some of these infections, especially cryptococcosis. Autopsy remains an important tool for quality assurance in health systems and further assists in guidelines implementation in public health systems.

Infections of the CNS are uncommon and life-threatening. The high mortality rate is thought to be related to the delays in diagnosis and inappropriate treatment.^5^ Of the 300 virulent fungal species to human, only about 10%–15% of these usually produce systemic/CNS mycosis.^4,7^ Opportunistic fungi are *Aspergillus fumigatus, Candida albicans, Cryptococcus neoformans* and *Rhizopus arrhizus*.^4,8^ It is important to note that these infections do not provide long-term immunity to the patients, and hence, relapses are noted.

The CNS is quite resilient to fungal infections, and its mode of infection is usually via haematogenous spread/seeding.^9^ However, spread from a parameningeal focus as well as direct inoculation at the time of trauma might also result in CNS disease.^9^ Disseminated mycosis is often associated with CNS involvement.^7^ The most common CNS fungal infection worldwide is cryptococcal meningoencephalitis.^7^ It is estimated that between 67% and 84% of patients with invasive cryptococcosis develop CNS mycosis, 3%–64% develop invasive candidiasis and 12% develop mucormycosis.^7^ The host defences and virulence of the infecting organism play a vital role in determining the signs and symptoms of CNS infections.^5,9,10^ In this study, the most common causative agent is cryptococcal infection, followed by candidiasis and mucormycosis in keeping with the existing literature. This trend is widely accepted and understandable in our setting, since South Africa is mostly affected by the HIV/AIDS epidemic with cryptococcal infection being the second most common AIDS-defining infection after tuberculosis. This is despite the extensive roadshows and guidelines by Nelesh et al. (2019) who advocate for the easiest way of monitoring and diagnosing this lethal infection using serum cryptococcal antigen from the blood taken for CD4 count on HIV infection.^11^

Despite the definite fungal organism identification, they all present with the same clinical features.^8^ The classic symptom triad of fever, neck stiffness and altered mental status is present in only a minority of patients.^12^ The clinical presentation ranges from meningoencephalitis, brain abscess and infarction to stroke.^3,4^ The clinical presentation and/or pathology of the lesions are usually determined by the morphology and the size of the organism.^9^ *Cryptococcus* and *Candida* enter the capillaries and subarachnoid spaces, causing meningitis and subpial ischemic lesions; *Candida* enter small blood vessels and cause local necrotic lesions and abscess; while mucormycetes penetrate large blood vessels and give rise to large infarcts and eventually strokes.^7,9^

Given the lack of specific symptoms or physical findings, the diagnosis of meningitis is based on the analysis of cerebrospinal fluid (CSF) in the absence of clear contraindications.^12,13^ CSF fluid analysis can help predict a bacterial, viral, or fungal cause for meningitis.^12^ CSF should be sent for culture special CSF testing for fungal (e.g. cryptococcal antigen, fungal culture).^8,12,14^ The CSF cryptococcal capsular polysaccharide antigen (CrAg) assay is both sensitive and specific for chronic meningitis (CM)^10^ and is also useful for prognostication. Fungal cultures are positive in more than 95% of *Cryptococcus neoformans* cases and in 66% of candidal meningitis cases while *Zygomycetes* are less likely to be culture positive.^8,14^

In this study, serum and CSF CrAg were only performed on two cases which were positive. Blood investigation was performed on the case with candidiasis which showed candidaemia.

A CT scan of the brain is routinely performed before lumbar puncture.^13^ Guidelines from the Infectious Disease Society of America (IDSA) recommend that cranial imaging before lumbar puncture be restricted to certain high-risk patients.^13^ Due to the high suspicion of metastatic disease, an MRI was only performed in case 4. While the absence of HIV infection in this case might have made it a less likely diagnosis, prolonged steroid therapy that the patient was on for MG rendered him immunocompromised. Therefore, there should have been a high index of suspicion for opportunistic infection. Moreover, cryptococcal infection has been described in the immunocompetent individual. In a recently published study by Wu et al.,^15^ fungal infection was not an antemortem diagnostic consideration which could have been attributed to the non-specific clinical features and low index of suspicion by the clinicians.

Brain biopsy is not usually routinely performed for histopathological diagnosis due to its invasive nature. It is for this reason that most of these cases are diagnosed at autopsy, especially in the challenging cases. The association of HIV infection and tuberculosis is high due to the nature of the HIV/AIDS epidemic in sub-Saharan Africa, especially the Southern African Development Community (SADC). This association has also contributed to a low index of suspicion for fungal infection of CNS; instead, most of these cases are diagnosed as disseminated tuberculosis. This further contributes towards a high rate of misdiagnosis, and therefore, wrong treatment is given to patients.

The gross brain features in CNS fungal infections are increased in weight and size with features of raised intracranial pressure (widened sulci and flattened gyri) and subsequent herniation. Some of the cases may present with surface exudate and engorged blood vessels. Mucormycosis shows liquefactive necrosis while cryptococcal infection demonstrates microcystic structures.

Cases 2–3 showed microcysts with necrosis of thalamus. Case 4 showed multiple well-circumscribed soft lesions which were worrisome for malignant disease, hence the metastatic work-up of the patient.

Microscopically, cryptococcal infection shows pauci-reactive and reactive pattern depending on the immune status of the patient. The immunocompetent patients are able to mount response; therefore, granulomatous inflammation becomes the most common inflammatory reaction pattern in these cases.^16–19^ The immunocompromised patients usually have mild inflammation or nothing at all. The inflammation presents with a soap bubble appearance as seen in all four of our cases in this study. Mucormycoses are aggressive as they present with angioinvasion and surrounding necrosis which was evident in case 5. Candidiasis may show acute suppurative inflammation with associated granulomatous inflammation response as noted in this study.^18^

While cryptococcal infection is the most common cause of CNS fungal infection as evidenced in this study, the species identified by Wu and colleagues were *Aspergillus, Blastomyces* and *Candida*.^15^ More cases of *Aspergillus* have been described recently; however, *Blastomyces* is very rare. This further highlights the importance of autopsy in identification of these fungal infections, including rare species.

Deaths caused by CNS fungal infection may be more common than previously appreciated. While it may seem easy to clinically diagnose the indistinguishable nature of these infections, this should prompt the clinicians to have a high index of suspicion in these cases. Furthermore, in the sub-Saharan Africa and/or Southern African context with high prevalence of HIV/AIDS, forensic and anatomical pathologists should also actively look for these infections during autopsy.

Finally, this study was retrospective, and therefore, autopsies were performed at different times for diagnostic purposes without research endeavour in mind. This contributed in clinical information missing such as microbiological analysis and radiological study including gross pictures in some of the cases. Notwithstanding this study period being 9 years, the sample was very small, and therefore not of any statistical significance.

## Conclusion

Despite advances in diagnosis and therapeutic management, fungal infections of the CNS are still difficult to treat, and therefore have a poor prognosis. A greater awareness of these infections might enable early initiation of appropriate therapy, resulting in improved outcomes. Furthermore, this study highlights the importance of autopsy as part of the clinical audit and/or aids in determining the causes of death in clinically challenging cases.

## References

[1] Cho TA. Management of Acute, recurrent, and chronic meningitides in adults. Neurol Clin NA 2019; 28(4): 1061–1088. 10.1016/j.ncl.2010.03.023 20816277

[2] Mathur M Johnson CE. Fungal infections of the central nervous system fungal infection abscess central nervous system meningitis cerebritis. Neuroimaging Clin NA 2005; 22(4): 609–632. 10.1016/j.nic.2012.04.004 23122259

[3] Schwartz S Kontoyiannis DP Harrison T , et al. Advances in the diagnosis and treatment of fungal infections of the CNS. Lancet Neurol 2019; 17(4): 362–372. 10.1016/S1474-4422(18)30030-9 29477506

[4] Raman Sharma R . Fungal infections of the nervous system: current perspective and controversies in management. Int J Surg 2010; 8(8): 591–601. 20673817 10.1016/j.ijsu.2010.07.293

[5] John R Hirsch N. Central nervous system infections in intensive care patients. Anaesth Intensive Care Med 2019; 16(4): 161–164.

[6] Zueter AM Zaiter A. Infectious meningitis. Clin Microbiol Newsl 2019; 37(6): 43–51.

[7] Góralska K Blaszkowska J Dzikowiec M. Neuroinfections caused by fungi. Infection 2018; 46(4): 443–459. 29785613 10.1007/s15010-018-1152-2 PMC6096918

[8] Badiee P Alborzi A. Assessment of a real-time PCR method to detect human non-cryptococcal fungal meningitis. Arch Iran Med 2011; 14(6): 381–384. 22039841

[9] Sethi PK Khanna L Batra A , et al. Central nervous system fungal infections: observations from a large tertiary hospital in northern India. Clin Neurol Neurosurg 2012; 114(9): 1232–1237. 22464435 10.1016/j.clineuro.2012.03.007

[10] Baldwin KJ Avila JD. Diagnostic approach to chronic meningitis. Neurol Clin NA 2019; 36(4): 831–849. 10.1016/j.ncl.2018.06.004 30366558

[11] Govender NP Meintjes G Mangena P , et al. Southern African HIV Clinicians Society guideline for the prevention, diagnosis and management of cryptococcal disease among HIV-infected persons: 2019 update. South Afr J HIV Med 2019; 20(1): 1030–1016. 10.4102/sajhivmed.v20i1.1030 PMC7081625 32201629

[12] Dorsett M. Diagnosis and treatment of central nervous system infections in the emergency. Emerg Med Clin NA 2019; 34(4): 917–942. 10.1016/j.emc.2016.06.013 PMC5082707 27741995

[13] Honda H Warren DK. Central nervous system infections: meningitis and brain abscess. Infect Dis Clin NA 2019; 23(3): 609–623. 10.1016/j.idc.2009.04.009 19665086

[14] O’Leary RA Einav S Leone M , et al. Management of invasive candidiasis and candidaemia in critically ill adults: expert opinion of the European society of anaesthesia intensive care scientific subcommittee. J Hosp Infect 2018; 98(4): 382–390. 29222034 10.1016/j.jhin.2017.11.020

[15] Wu G Liu Y Bulakhtina E , et al. Forensic neuropathologic phenotypes of fungal central nervous system infections: a case series. Am J Forensic Med Pathol 2021; 42(4): 383–386. 34354012 10.1097/PAF.0000000000000704

[16] Klock C Cerski M Goldani LZ. Histopathological aspects of neurocryptococcosis in HIV-infected patients: autopsy report of 45 patients. Int J Surg Pathol 2009; 17(6): 444–448. 18611927 10.1177/1066896908320550

[bibr17-2050313X221122419] Shibuya K Hirata A Omuta J , et al. Granuloma and cryptococcosis. J Infect Chemother 2005; 11(3): 115–122. 15990974 10.1007/s10156-005-0387-x

[bibr18-2050313X221122419] Shibuya K Coulson WF Wollman JS , et al. Histopathology of cryptococcosis and other fungal infections in patients with acquired immunodeficiency syndrome. Int J Infect Dis 2001; 5(2): 78–85. 11468102 10.1016/s1201-9712(01)90030-x

[19] Sing Y Ramdial PK. Cryptococcal inflammatory pseudotumors. Am J Surg Pathol 2007; 31(10): 1521–1527. 17895752 10.1097/PAS.0b013e318040ad0a

